# Drug-induced autoimmune-like hepatitis: A disproportionality analysis based on the FAERS database

**DOI:** 10.1371/journal.pone.0317680

**Published:** 2025-02-06

**Authors:** Wangyu Ye, Yuan Ding, Meng Li, Zhihua Tian, Shaoli Wang, Zhen Liu

**Affiliations:** Guang’anmen Hospital, China Academy of Chinese Medical Sciences, Beijing, China; Hong Kong Baptist University, HONG KONG

## Abstract

**Background:**

Drug-induced autoimmune-like hepatitis (DI-ALH) is a potentially life-threatening condition that can lead to acute liver failure and necessitate liver transplantation. While the association between certain drugs and DI-ALH has been documented, a comprehensive analysis of drug-related signals in a large, real-world pharmacovigilance database is lacking. This study aimed to systematically identify drugs linked to DI-ALH by analyzing adverse event reports from the U.S. Food and Drug Administration’s (FDA) Adverse Event Reporting System (FAERS) database.

**Methods:**

We searched the FAERS database for the term "autoimmune hepatitis" and extracted DI-ALH reports from the first quarter of 2004 to the first quarter of 2024. Positive signal drugs were identified using Proportional Reporting Ratio (PRR), Reporting Odds Ratio (ROR), Bayesian Confidence Propagation Neural Network (BCPNN), and Empirical Bayesian Geometric Mean (EBGM). To confirm a significant drug-adverse event association, each method had to meet predefined thresholds: for PRR and ROR, values were considered significant if the lower 95% confidence interval (CI) was greater than 1 and at least three reports were identified; for BCPNN, an Information Component (IC025) greater than 0 indicated a signal; for EBGM, a value greater than 2 for the lower 95% confidence interval (EBGM05) was used to denote a positive signal.

**Results:**

A total of 5,723 DI-ALH reports were extracted from the FAERS database. Disproportionality analysis identified 50 drugs with strong associations to DI-ALH, with biologics, statins, antibiotics, and antiviral drugs representing the most common categories. Among these, nitrofurantoin (ROR 94.79, CI 78.53–114.41), minocycline (ROR 77.82, CI 65.09–93.05), and nivolumab (ROR 47.12, CI 15.06–147.39) exhibited the strongest signals. Additionally, several previously unreported drugs, including mesalazine, aldesleukin, onasemnogene abeparvovec-xioi, and nefazodone, were identified as having strong associations with DI-ALH. These findings were consistent across all four signal detection methods, further validating the robustness of the associations.

**Conclusion:**

This study provides a comprehensive assessment of drugs associated with DI-ALH through a rigorous analysis of the FAERS database using multiple signal detection methods. By identifying both well-known and previously underreported drugs, this study contributes to a more complete understanding of drug-induced liver injury. The findings have important implications for pharmacovigilance strategies and clinical risk assessment. However, limitations inherent in the FAERS database, such as underreporting and the potential for reporting bias, should be considered. Further clinical validation is warranted to confirm these associations.

## Introduction

Drug-induced liver injury (DILI) is liver damage caused by drugs or their metabolites, occupying a significant position in the spectrum of liver diseases and posing one of the most serious challenges in drug adverse reactions. The pathogenesis of DILI is diverse, involving multiple mechanisms such as direct toxicity, immune-mediated reactions, bile duct obstruction, and mitochondrial dysfunction [1–3]. The clinical phenotypes of DILI are complex and varied, presenting as acute hepatocellular injury, cholestasis, or a mixed pattern thereof. In rare cases, DILI can progress to chronic liver disease, with some patients exhibiting distinct clinical phenotypes [4, 5]. In patients with drug-induced liver injury (DILI), it is sometimes possible to observe pathological and laboratory findings with autoimmune characteristics, a condition known as drug-induced autoimmune-like hepatitis (DI-ALH). DI-ALH represents a specific subtype of DILI, characterized by clinical, biochemical, and histological features that closely resemble those of autoimmune hepatitis (AIH) [6, 7]. Laboratory tests often reveal markedly elevated serum transaminases (ALT and AST), increased immunoglobulin G (IgG), and positive autoimmune-related antibodies (such as antinuclear antibodies ANA and anti-smooth muscle antibodies SMA). Some patients may progress to acute liver failure, characterized by coagulopathy and hepatic encephalopathy, posing a life-threatening condition [8–10].

A prospective study conducted between 1997 and 2000 investigated the incidence of DILI. The study results showed an annual crude incidence rate of 13.9 cases per 100,000 residents for DILI. Additionally, the study indicated that the actual incidence of DILI in the French population might be 16 times higher than reports voluntarily submitted to French regulatory agencies [11]. Another prospective study conducted in Iceland during 2010 to 2011 investigated the incidence of DILI, with a crude annual incidence rate of 19.1 cases per 100,000 people [6]. In recent years, with the increasing incidence of DILI, the incidence of DI-ALH has also been rising, accounting for approximately 6% to 22% of DILI cases [12, 13]. Among 261 patients diagnosed with autoimmune hepatitis (AIH), 24 cases (9.2%) were retrospectively identified as DI-ALH [14]. In the DILI registry database in Spain, approximately 1.2% of patients experienced recurrent DILI episodes triggered by different drugs. These patients were more likely to exhibit features of AIH during the second episode [15]. Despite the relatively low incidence rate of DI-ALH, its risk of causing acute liver injury, tendency towards chronicity, and potential for recurrence pose significant challenges and potential hazards in clinical management, potentially endangering patients’ lives.

However, the diagnosis of DI-ALH currently faces many challenges, primarily because it shares multiple similarities with idiopathic AIH. The overlap in clinical presentations, biochemical markers, and histological features poses a significant challenge. Coupled with the lack of specific biomarkers, these factors make differential diagnosis exceptionally complex [13, 16, 17]. As Castiella et al. emphasize, the presence of a definable drug-related cause should theoretically distinguish DIAIH from AIH. However, this distinction is complicated in practice, as retrospective studies often fail to account for cases where drug-induced hepatitis is misclassified as AIH. Such diagnostic uncertainty is compounded by the fact that both conditions can present with similar serological markers, such as positive antinuclear antibodies and smooth muscle antibodies [18]. For example, in a cohort study of 261 patients, 83% of DIAIH patients and 70% of AIH patients tested positive for antinuclear antibodies, while 50% of DIAIH patients and 45% of AIH patients were positive for smooth muscle antibodies. This overlap further blurs the lines between the two conditions and complicates clinical decision-making [19]. There is currently a lack of widely accepted unified criteria for assessing the causality between certain drugs and DI-ALH, leading to clinical discrepancies [8]. Given the rising global incidence of DILI and DI-ALH, understanding the risk factors for DI-ALH is crucial for early identification, timely discontinuation of the causative drugs, and the development of targeted treatment strategies. This study provides valuable insights into the pharmacovigilance of DI-ALH and its associated drugs, addressing the increasing clinical need to enhance diagnosis, treatment, and patient safety.

The U.S. Food and Drug Administration Adverse Event Reporting System (FAERS) database contains spontaneous adverse event reports from global sources, encompassing diverse drugs and patient populations. This diversity addresses the limitations of traditional research regarding sample representativeness. Moreover, the FAERS database is continually updated, enabling real-time drug safety monitoring and facilitating the early detection of hepatotoxicity signals in both newly marketed and long-established drugs [20]. In this study, adverse event reports related to DI-ALH were extracted and analyzed using disproportionality analysis and signal detection methods, which leverage the database’s large-scale and diverse dataset to identify statistically significant associations between specific drugs and DI-ALH.

## Methods

### Data source

To obtain pharmacovigilance information related to DI-ALH, we conducted a retrospective analysis of the publicly available FAERS database (https://fis.fda.gov/extensions/FPD-QDE-FAERS/FPD-QDE-FAERS.html). The FAERS database compiles reports submitted by healthcare professionals, consumers, and manufacturers, providing a robust resource for monitoring drug safety. With its large-scale dataset and real-time reporting capabilities, FAERS enables the detection of rare and severe adverse events such as DI-ALH, supporting regulatory assessments and clinical risk evaluations [21]. In this study, we retrieved all adverse event reports related to DI-ALH from the FAERS database from the first quarter of 2004 to the first quarter of 2024, and we extracted, organized, and analyzed the data using R Studio software version 4.3.3.

### Identification of DI-ALH report and drugs

Using MedDRA to standardize adverse event information in the FAERS database is a key step in ensuring the consistency and accuracy of drug safety monitoring data. Adverse event (AE) information extracted from the FAERS database is standardized and coded according to the Preferred Terms (PT) in the Medical Dictionary for Regulatory Activities (MedDRA, version 26.1). In this study, the PT field in MedDRA was searched for "drug-induced autoimmune-like hepatitis" (MedDRA code: 10003827) to retrieve all adverse event reports of AIH. The Medex_UIMA_1.3.8 system was used to standardize drug names in the adverse event reports, and the top 50 drugs associated with AIH were identified.

### DI-ALH signal analysis method

Disproportionality analysis is a widely used statistical method in pharmacovigilance to evaluate the association between specific drugs and adverse reactions in drug adverse event reports. In this study, signal detection methods such as Proportional Reporting Ratio (PRR), Reporting Odds Ratio (ROR), Bayesian Confidence Propagation Neural Network (BCPNN), and Empirical Bayes Geometric Mean (EBGM) were used to assess the association between drugs and autoimmune hepatitis (AIH). PRR and ROR are frequentist measures of disproportionality, with a signal considered positive if the lower 95% confidence interval (CI) for ROR exceeds 1 and at least three reports of the adverse event are present. A PRR > 2, a chi-squared value > 4, and at least three reports also indicate a potential signal. Larger values of PRR and ROR signify stronger associations. BCPNN and EBGM are Bayesian methods that account for reporting biases and variability in sample sizes. BCPNN uses the information component (IC), with a signal defined by IC025 (the lower bound of the 95% CI for IC) > 0. Higher IC values indicate stronger associations. EBGM evaluates associations through an empirical Bayes framework, with a signal indicated by EBGM05 > 2. These Bayesian methods offer greater robustness, particularly in handling data sparsity and reporting heterogeneity. The calculation formulas and signal detection criteria are detailed in Table 1.

**Table 1:** Summary of four signal detection algorithms.

Method	Formula	Threshold
ROR	$\text{ROR}=\frac{\text{a}/\text{c}}{\text{b}/\text{d}}$	a ≥ 3
	$\text{SE}\left(\text{lnROR}\right)=\sqrt{\frac{1}{\text{a}}+\frac{1}{\text{b}}+\frac{1}{\text{c}}+\frac{1}{\text{d}}}$	
	95%CI = e^ln(ROR)±1.96se^	95%CI (lower limit) > 1
PRR	$\text{PRR}=\frac{\text{a}/\left(\text{a}+\text{b}\right)}{\text{c}/\left(\text{c}+\text{d}\right)}$	a ≥ 3
	$\text{SE}\left(\text{lnPRR}\right)=\sqrt{\frac{1}{\text{a}}-\frac{1}{\text{a}+\text{b}}+\frac{1}{\text{c}}-\frac{1}{\text{c}+\text{d}}}$	
	95%CI = e^ln(PRR)±1.96se^	95%CI (lower limit) > 1
BCPNN	$\text{IC}={\text{log}}_{2}\frac{\text{p}\left(\text{x},\text{y}\right)}{\text{p}\left(\text{x}\right)\text{p}\left(\text{y}\right)}={\text{log}}_{2}\frac{\text{a}\left(\text{a}+\text{b}+\text{c}+\text{d}\right)}{\left(\text{a}+\text{b}\right)\left(\text{a}+\text{c}\right)}$	IC025>0
	$\text{E}\left(\text{IC}\right)={\text{log}}_{2}\frac{\left(\text{a}+\gamma 11\right)\left(\text{a}+\text{b}+\text{c}+\text{d}+\alpha \right)\left(\text{a}+\text{b}+\text{c}+\text{d}+\beta \right)}{\left(\text{a}+\text{b}+\text{c}+\text{d}+\gamma \right)\left(\text{a}+\text{b}+\alpha 1\right)\left(\text{a}+\text{c}+\beta 1\right)}$	
	$\begin{array}{ll} \text{V}\left(\text{IC}\right) & =\frac{1}{{\left(\text{ln}2\right)}^{2}}[\frac{\left(\text{a}+\text{b}+\text{c}+\text{d}\right)-\text{a}+\gamma -\gamma 11}{\left(\text{a}+\gamma 11\right)\left(1+\text{a}+\text{b}+\text{c}+\text{d}+\gamma \right)}\\ & +\frac{\left(\text{a}+\text{b}+\text{c}+\text{d}\right)-\left(\text{a}+\text{b}\right)+\text{a}-\alpha 1}{\left(\text{a}+\text{b}+\alpha 1\right)\left(1+\text{a}+\text{b}+\text{c}+\text{d}+\alpha \right)}\\ & +\frac{\left(\text{a}+\text{b}+\text{c}+\text{d}+\alpha \right)-\left(\text{a}+\text{c}\right)+\beta -\beta 1}{\left(\text{a}+\text{b}+\beta 1\right)\left(1+\text{a}+\text{b}+\text{c}+\text{d}+\beta \right)}] \end{array}$	
	$\gamma =\gamma 11\frac{\left(\text{a}+\text{b}+\text{c}+\text{d}+\alpha \right)\left(\text{a}+\text{b}+\text{c}+\text{d}+\beta \right)}{\left(\text{a}+\text{b}+\alpha 1\right)\left(\text{a}+\text{c}+\beta 1\right)}$	
	$\text{IC}-2\text{SD}=\text{E}\left(\text{IC}\right)-2\sqrt{\text{V}\left(\text{IC}\right)}$	
EBGM	$\text{EBGM}=\frac{\text{a}\left(\text{a}+\text{b}+\text{c}+\text{d}\right)}{\left(\text{a}+\text{c}\right)\left(\text{a}+\text{b}\right)}$	EBGM05>2
	$\text{SE}\left(\text{lnEBGM}\right)=\sqrt{\frac{1}{\text{a}}+\frac{1}{\text{b}}+\frac{1}{\text{c}}+\frac{1}{\text{d}}}$	
	95%CI = e^ln(EBGM)±1.96se^	

## Results

### Basic information on DI-ALH adverse event report

**Fig 1:**
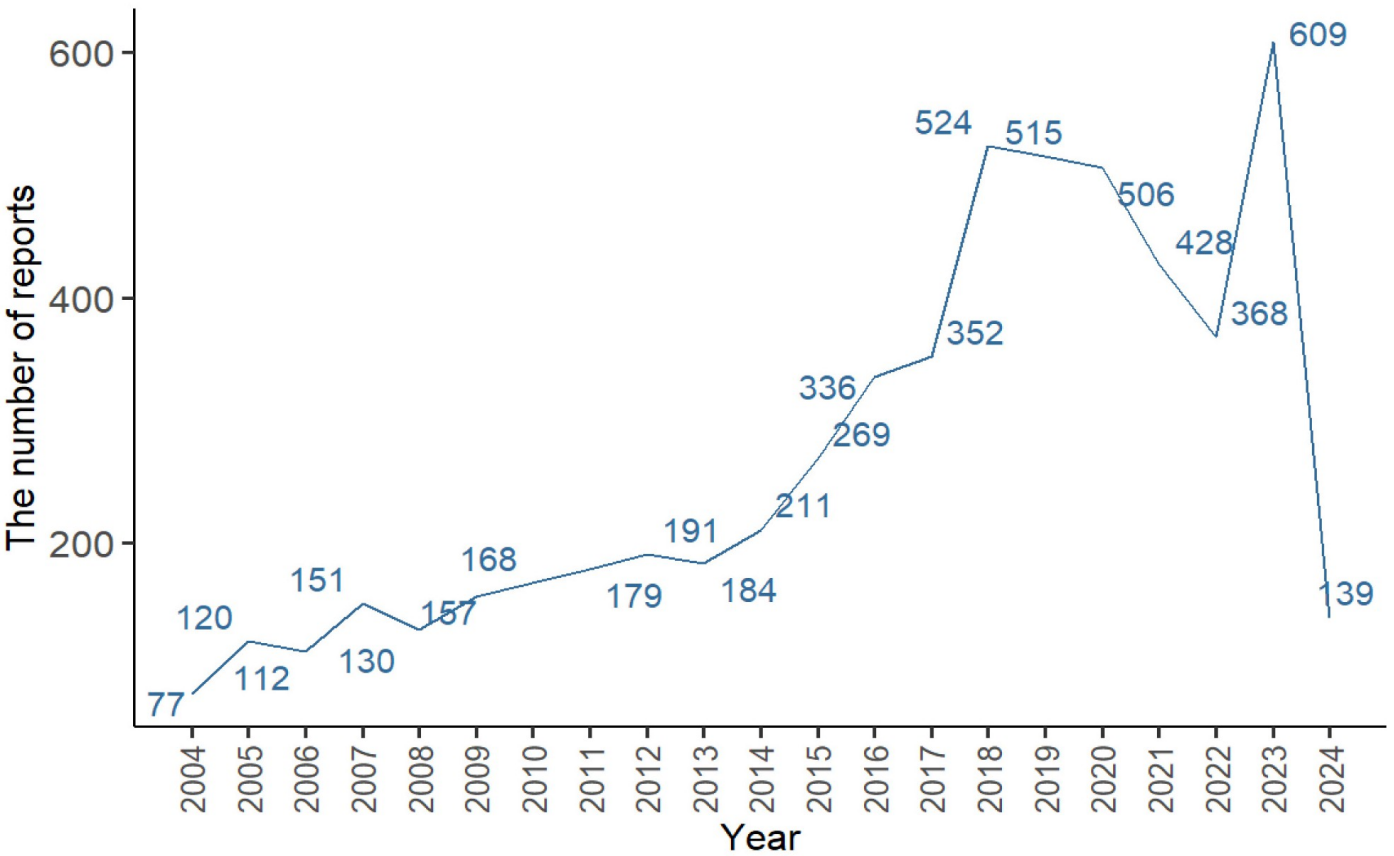
Number of reported cases of DI-ALH from Q1 2004 to Q1 2024.

From the first quarter of 2004 to the first quarter of 2024, we extracted a total of 5723 DI-ALH adverse event reports from the FAERS database. We performed statistical analysis and visualization of adverse event report data submitted from 2004 to 2024. The results showed a fluctuating upward trend in the number of adverse event reports related to drug-induced AIH (Fig 1). Within this trend, the number of reports peaked in 2023, with a total of 609 cases reported. Among all DI-ALH cases (Table 2), the incidence was highest in the 45–65 age group (32.50%), followed by the 19–45 age group (17.46%), under 19 years (4.25%), 65–75 years (14.70%), and over 75 years (8.25%). In terms of gender, the incidence was higher in females (61.12%) than in males (28.67%). Most of the submitted reports came from the United States (26.04%), followed by Germany (6.92%), Japan (5.61%), France (5.56%), and the United Kingdom (4.67%), among other countries. Among the extracted reports, the most common clinical outcome was other serious—Important Medical Events (54.38%), followed by hospitalization (32.09%), death (5.59%), life threatening (5.04%), disability (2.32%), required intervention to Prevent Permanent Impairment/Damage (0.55%), and congenital anomaly (0.04%).

**Table 2:** Clinical characteristics of patients with DI-ALH.

variable	Total
**sex**	
female	3498 (61.12)
male	1641 (28.67)
unknown	584 (10.20)
**Median Age**	55.00 (42.00,67.00)
**Age**	
<19	243 (4.25)
19~45	999 (17.46)
45~65	1860 (32.50)
65~75	841 (14.70)
> = 75	472 (8.25)
unknow	1308 (22.86)
**Weight**	70.00 (58.00,84.00)
**Reporter**	
Physician	2520 (44.03)
Other health-professional	1153 (20.15)
Consumer	898 (15.69)
Pharmacist	858 (14.99)
unknown	266 (4.65)
Lawyer	27 (0.47)
Registered Nurse	1 (0.02)
**Reported countries**	
other	2931 (51.21)
United States	1490 (26.04)
Germany	396 (6.92)
Japan	321 (5.61)
France	318 (5.56)
United Kingdom	267 (4.67)
**Outcomes**	
other serious	4360 (54.38)
hospitalization	2573 (32.09)
death	448 (5.59)
life threatening	404 (5.04)
disability	186 (2.32)
required intervention to Prevent Permanent Impairment/Damage	44 (0.55)
congenital anomaly	3 (0.04)
**Time to onset**	86.00 (25.00,313.00)

**Fig 2:**
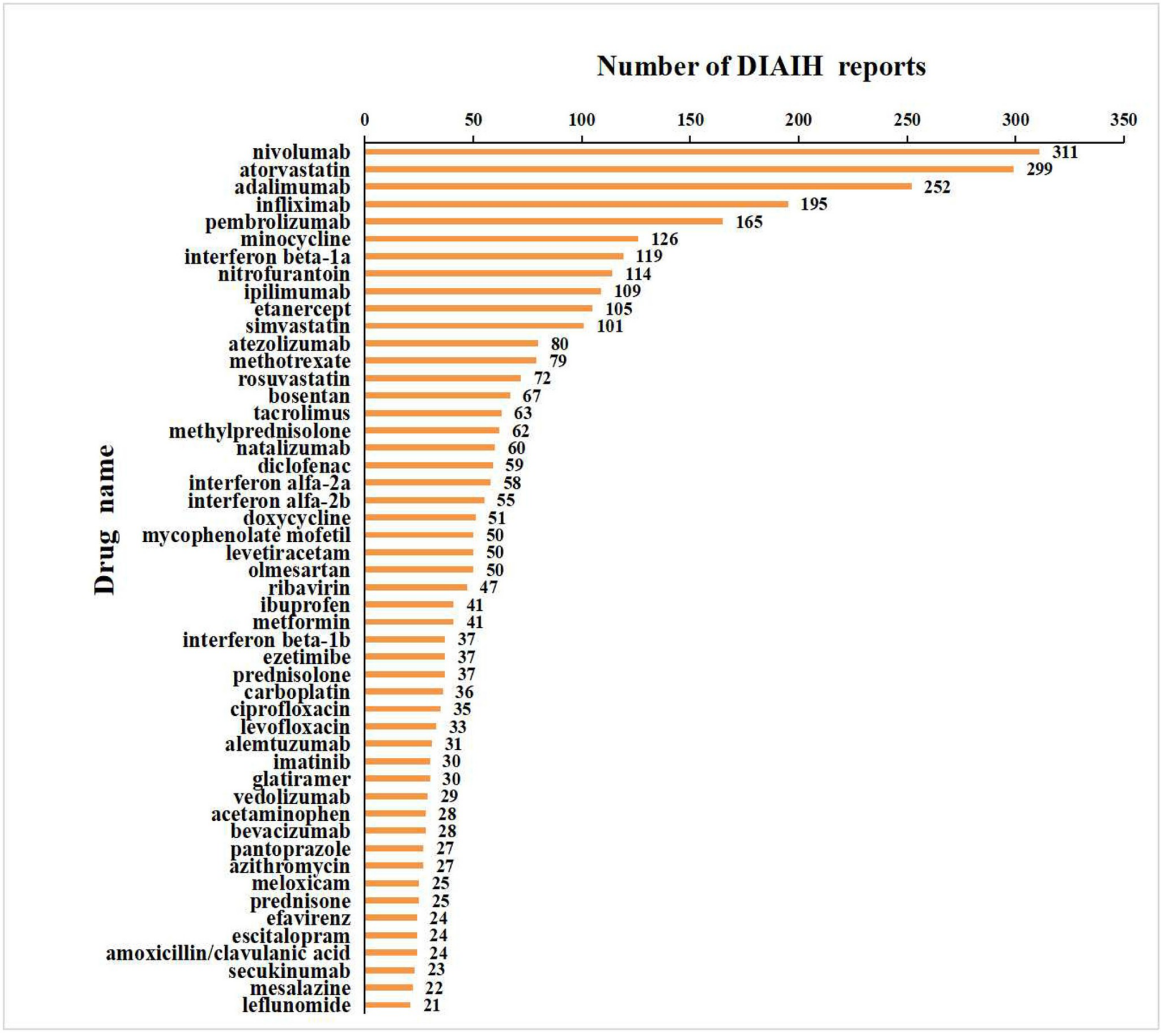
Top 50 drugs with the highest number of reported DI-ALH.

Based on the frequency of adverse events, we ranked the top 50 drugs associated with AIH (Fig 2). Nivolumab (311 cases) was the most frequently implicated drug in AIH, followed by atorvastatin (299 cases), adalimumab (252 cases), infliximab (195 cases), pembrolizumab (165 cases), minocycline (126 cases), interferon beta-1a (119 cases), and nitrofurantoin (114 cases). This study investigated the top 50 drugs leading to AIH, including monoclonal antibodies, nonsteroidal anti-inflammatory drugs (NSAIDs), antibiotics, antiviral drugs, immunosuppressants, glucocorticoids, lipid-lowering drugs, antidepressants, and hypoglycemic agents.

### Disproportionality analysis

In this study, we used four signal detection methods—PRR, ROR, BCPNN, and EBGM—to assess the strength of association between drugs and DI-ALH. We ranked the signal strength based on ROR values and listed the top 50 drugs with the strongest signals in Table 3. Most of these drugs are monoclonal antibodies, statins, antibiotics, and antiviral drugs. Nitrofurantoin (ROR 94.79, CI 78.53–114.41) had the strongest signal, followed by minocycline (ROR 77.82, CI 65.09–93.05), elbasvir (ROR 47.12, CI 15.06–147.39), fluvastatin (ROR 35.98, CI 22.59–57.29), mogamulizumab (ROR 22.88, CI 11.87–44.11), aldesleukin (ROR 22.06, CI 8.25–59.01), propylthiouracil (ROR 21.75, CI 8.13–58.18), hydralazine (ROR 20.44, CI 13.3–31.42), ipilimumab (ROR 20.34, CI 16.82–24.6), and cemiplimab(ROR 17.23, CI 8.94–33.19).

**Table 3:** Top 50 drugs for signal strength.

Drug	Case Reports	ROR (95% CI)	PRR (95% CI)	chisq	IC (IC025)	EBGM (EBGM05)
nitrofurantoin	114	94.79 (78.53, 114.41)	92.05 (77.16, 109.81)	10065.79	6.5 (6.23)	90.24 (77.09)
minocycline	126	77.82 (65.09, 93.05)	75.98 (63.69, 90.64)	9120.48	6.22 (5.96)	74.33 (64)
elbasvir	3	47.12 (15.06, 147.39)	46.42 (15.19, 141.87)	133.31	5.54 (4.11)	46.4 (17.87)
fluvastatin	18	35.98 (22.59, 57.29)	35.57 (22.66, 55.83)	603.14	5.15 (4.49)	35.47 (24.03)
mogamulizumab	9	22.88 (11.87, 44.11)	22.72 (11.9, 43.38)	186.68	4.5 (3.61)	22.69 (13.1)
aldesleukin	4	22.06 (8.25, 59.01)	21.91 (8.22, 58.38)	79.81	4.45 (3.18)	21.9 (9.61)
propylthiouracil	4	21.75 (8.13, 58.18)	21.61 (8.11, 57.58)	78.59	4.43 (3.16)	21.59 (9.48)
hydralazine	21	20.44 (13.3, 31.42)	20.31 (13.2, 31.26)	384.34	4.34 (3.73)	20.24 (14.13)
ipilimumab	109	20.34 (16.82, 24.6)	20.22 (16.62, 24.6)	1953.7	4.31 (4.04)	19.85 (16.93)
cemiplimab	9	17.23 (8.94, 33.19)	17.14 (8.98, 32.73)	136.59	4.1 (3.2)	17.11 (9.89)
albendazole	5	16.88 (7, 40.66)	16.79 (6.95, 40.56)	74.2	4.07 (2.91)	16.78 (8.04)
nivolumab	311	16.24 (14.48, 18.21)	16.17 (14.38, 18.19)	4186.17	3.94 (3.77)	15.34 (13.94)
emtricitabine	3	15.95 (5.13, 49.61)	15.87 (5.09, 49.46)	41.79	3.99 (2.57)	15.86 (6.14)
metreleptin	5	15.57 (6.46, 37.5)	15.5 (6.42, 37.44)	67.76	3.95 (2.79)	15.48 (7.42)
efavirenz	24	15.44 (10.33, 23.08)	15.37 (10.39, 22.75)	321.14	3.94 (3.37)	15.31 (10.94)
meloxicam	25	14.33 (9.67, 21.24)	14.27 (9.64, 21.12)	307.22	3.83 (3.27)	14.21 (10.22)
atezolizumab	80	12.67 (10.16, 15.81)	12.63 (10.18, 15.67)	844.66	3.64 (3.32)	12.46 (10.36)
pembrolizumab	165	12.49 (10.69, 14.58)	12.44 (10.63, 14.55)	1686.89	3.6 (3.38)	12.11 (10.64)
atorvastatin	299	11.94 (10.63, 13.42)	11.9 (10.58, 13.39)	2831.22	3.5 (3.33)	11.33 (10.28)
simvastatin	101	11.47 (9.42, 13.97)	11.43 (9.4, 13.9)	944.77	3.49 (3.21)	11.25 (9.54)
olmesartan	50	11.46 (8.67, 15.14)	11.42 (8.68, 15.03)	471.36	3.5 (3.1)	11.33 (8.97)
fenofibrate	12	11.45 (6.49, 20.2)	11.42 (6.47, 20.16)	113.84	3.51 (2.72)	11.39 (7.09)
doxycycline	51	10.76 (8.17, 14.19)	10.73 (8.16, 14.12)	446.11	3.41 (3.02)	10.64 (8.45)
amoxicillin/clavulanic acid	24	10.55 (7.06, 15.77)	10.52 (7.11, 15.57)	205.96	3.39 (2.82)	10.48 (7.49)
interferon alfa-2b	55	10.01 (7.67, 13.06)	9.98 (7.59, 13.13)	440.17	3.31 (2.93)	9.89 (7.92)
ezetimibe	37	9.56 (6.92, 13.21)	9.53 (6.96, 13.04)	280.87	3.24 (2.78)	9.48 (7.23)
alemtuzumab	31	8.72 (6.13, 12.42)	8.7 (6.11, 12.38)	210.25	3.11 (2.61)	8.66 (6.44)
nefazodone	3	8.46 (2.72, 26.28)	8.44 (2.71, 26.31)	19.67	3.08 (1.66)	8.44 (3.27)
cephalexin	11	8.17 (4.52, 14.77)	8.15 (4.53, 14.67)	68.91	3.02 (2.21)	8.14 (4.96)
ribavirin	47	8.12 (6.09, 10.82)	8.1 (6.04, 10.87)	290.22	3.01 (2.6)	8.04 (6.32)
methylprednisolone	62	8.09 (6.29, 10.39)	8.07 (6.25, 10.41)	379.88	3 (2.64)	7.99 (6.48)
pravastatin	16	7.7 (4.71, 12.59)	7.69 (4.71, 12.55)	92.85	2.94 (2.25)	7.67 (5.08)
ketoprofen	3	7.23 (2.33, 22.47)	7.22 (2.32, 22.5)	16.07	2.85 (1.43)	7.22 (2.8)
methimazole	4	7.17 (2.69, 19.13)	7.16 (2.69, 19.08)	21.18	2.84 (1.57)	7.15 (3.15)
indomethacin	6	7.14 (3.2, 15.92)	7.13 (3.19, 15.93)	31.59	2.83 (1.76)	7.12 (3.64)
acitretin	5	6.97 (2.9, 16.78)	6.96 (2.88, 16.81)	25.5	2.8 (1.64)	6.95 (3.34)
terbinafine	19	6.91 (4.4, 10.85)	6.9 (4.4, 10.83)	95.54	2.78 (2.15)	6.88 (4.72)
onasemnogene abeparvovec-xioi	4	6.7 (2.51, 17.87)	6.69 (2.51, 17.83)	19.33	2.74 (1.47)	6.68 (2.94)
avelumab	4	6.46 (2.42, 17.24)	6.45 (2.42, 17.19)	18.42	2.69 (1.42)	6.45 (2.84)
anastrozole	29	6.38 (4.43, 9.2)	6.37 (4.39, 9.24)	130.74	2.67 (2.15)	6.35 (4.67)
darunavir	7	5.88 (2.8, 12.35)	5.87 (2.79, 12.36)	28.26	2.55 (1.55)	5.87 (3.15)
mesalazine	22	5.74 (3.78, 8.73)	5.73 (3.8, 8.65)	85.66	2.51 (1.92)	5.71 (4.02)
pemetrexed	21	5.67 (3.69, 8.71)	5.66 (3.68, 8.71)	80.31	2.5 (1.89)	5.64 (3.94)
interferon alfa-2a	58	5.65 (4.36, 7.31)	5.64 (4.37, 7.28)	219.04	2.48 (2.11)	5.59 (4.5)
durvalumab	17	5.55 (3.45, 8.94)	5.54 (3.46, 8.87)	63.13	2.47 (1.8)	5.53 (3.71)
daclizumab	4	5.3 (1.99, 14.13)	5.29 (1.99, 14.09)	13.91	2.4 (1.14)	5.29 (2.33)
rosuvastatin	72	5.27 (4.18, 6.65)	5.26 (4.16, 6.65)	245.56	2.38 (2.05)	5.21 (4.29)
irbesartan	13	5.25 (3.04, 9.04)	5.24 (3.03, 9.07)	44.5	2.39 (1.63)	5.23 (3.32)
bosentan	67	5.14 (4.04, 6.54)	5.14 (4.06, 6.5)	220.58	2.35 (2)	5.09 (4.16)
lamivudine	17	5.05 (3.13, 8.12)	5.04 (3.15, 8.07)	54.88	2.33 (1.66)	5.03 (3.37)

## Discussion

DI-ALH is a specific type of DILI, classified under the category of indirect liver injury. The pathogenesis of DI-ALH is not yet fully elucidated. It is an immune-mediated inflammatory response in the liver triggered by specific drugs, involving complex immunological processes and drug-host interactions [7, 22, 23]. Although timely identification and discontinuation of the suspected drug are the primary measures for treating DI-ALH, there is still a lack of comprehensive understanding of the drugs that may induce autoimmune hepatitis. This study leverages large-scale real-world medication data from the FAERS database to identify drugs closely associated with DI-ALH. We found that although certain drugs in the FAERS database show a close association with DI-ALH, their package inserts do not explicitly list adverse reactions related to DI-ALH. This finding suggests potential limitations in current research and drug regulation. Our findings confirm the previously reported associations between DI-ALH and drugs such as statins, interferons, hydralazine, nitrofurantoin, minocycline, propylthiouracil, and meloxicam [24–28]. Additionally, our study identifies associations with drugs not previously reported in the literature, including mesalazine, aldesleukin, onasemnogene abeparvovec-xioi, and nefazodone.

DI-ALH represents a severe adverse reaction that demands attention in clinical practice. In our study, the incidence rate among females was significantly higher than in males, aligning with previous findings that autoimmune diseases are more prevalent in women [29, 30]. This gender disparity may be attributed to factors such as estrogen levels, incomplete X chromosome inactivation, and fetal microchimerism during pregnancy [31–33]. These mechanisms likely amplify immune dysregulation, predisposing women to autoimmune conditions. Age also plays a critical role in DI-ALH susceptibility. Our findings revealed a predominance of DI-ALH cases in middle-aged and elderly populations. This can be partially explained by the physiological changes associated with aging, including a decline in drug metabolism and immune function. Furthermore, middle-aged and elderly individuals are often burdened with multiple chronic conditions requiring long-term polypharmacy, which elevates the risk of drug interactions and adverse immune responses [34]. The interplay between gender and age underscores the complexity of DI-ALH pathogenesis.

Statins are currently the most commonly used lipid-lowering drugs, widely used for the prevention and treatment of cardiovascular diseases, though DILI related to statins is extremely rare. In this study, drugs such as fluvastatin, atorvastatin, simvastatin, pravastatin, and rosuvastatin are all positively associated with AIH, consistent with previous case reports and literature. Previous studies indicate a risk of inducing autoimmune diseases with long-term use of statins. The pathogenesis of statin-induced AIH may involve both immune and metabolic pathways. Statins have been shown to induce oxidative stress by promoting the production of reactive oxygen species (ROS) and lipid peroxidation, leading to mitochondrial dysfunction and cellular apoptosis. This process may release intracellular antigens into circulation, stimulating the production of autoantibodies. Additionally, statins can disrupt calcium homeostasis, leading to mitochondrial calcium overload and permeability transition pore (PTP) opening, which exacerbates hepatocyte injury. Genetic predispositions, including variations in cytochrome P450 enzymes and transporter genes, may further amplify susceptibility to liver injury in some patients [28, 35, 36]. While the precise immunological mechanisms remain unclear, it is hypothesized that statins may abnormally activate immune pathways, resulting in the expansion of autoreactive CD8+ T lymphocytes that attack liver tissue [37]. Evolocumab, as a non-statin lipid-lowering drug, has fewer reported cases associated with AIH. In clinical studies, there is a case of AIH in a patient receiving combined therapy of atorvastatin and evolocumab; research suggests that evolocumab may be a triggering factor for AIH, but further studies are needed to clarify this correlation [38].

Biologics are novel therapeutic drugs for various immune-mediated inflammatory diseases, widely used in conditions such as inflammatory bowel disease and rheumatoid arthritis, showing significant therapeutic efficacy and promising applications. However, contradictorily, increasing evidence from studies confirms the risk of inducing autoimmune diseases with biologics. These drugs may impact the immune system through complex mechanisms, sometimes leading to unexpected autoimmune reactions [39, 40]. With the widespread use of biologics, DI-ALH due to immune-modulating therapies is increasingly recognized. Monoclonal antibodies are the most common biologics, closely associated with immunogenic adverse reactions and liver injury [41, 42]. Among these, TNF inhibitors such as infliximab are frequently implicated, with reports highlighting autoimmune hepatitis as a recognized adverse effect [8]. The mechanisms underlying DI-ALH remain complex and multifaceted. Current evidence suggests that biologics may disrupt immune tolerance by interfering with regulatory pathways, such as CD4+CD25+ regulatory T-cell function, which is critical in maintaining immunological homeostasis. This disruption may predispose individuals to aberrant immune responses, culminating in liver-targeted autoimmunity [43]. Furthermore, genetic predispositions, including specific HLA alleles and pre-existing immunological markers, may contribute to the development of DI-ALH [44]. For instance, patients with underlying autoimmune diseases such as rheumatoid arthritis or inflammatory bowel disease may exhibit heightened susceptibility due to their existing immune dysregulation [39, 45].

Our study identified significant associations between antibiotics, antiviral drugs, NSAIDs, interferons, and AIH. Previous research has extensively demonstrated that nitrofurantoin, minocycline, hydralazines, and interferons can induce DI-ALH [46]. In recent years, NSAIDs like meloxicam and indomethacin have increasingly been linked to DI-ALH [24, 47, 48]. The combination of interferon and ribavirin is a classical antiviral treatment, historically widely used for chronic hepatitis C (HCV), but associated with many cases of AIH induction [49–51]. Matsumoto et al. reported the first case of liver dysfunction following Elbasvir and Grazoprevir treatment, characterized by positive serum anti-nuclear antibodies and elevated immunoglobulin G levels. Liver biopsy showed severe interface hepatitis with lymphocyte and plasma cell infiltration, clinically diagnosed as DI-ALH related to direct antiviral drugs [52]. There is currently no established link in the literature between drugs like entecavir, sofosbuvir, and daclatasvir with AIH, necessitating further investigation.

## Limitations

Although this study is based on real-world pharmacovigilance data, providing references for identifying drugs that may induce DI-ALH, it inevitably has some limitations. Firstly, the system relies on voluntary reporting, which may lead to underreporting or selective reporting of adverse events, thus affecting the integrity and representativeness of the data. Secondly, reports often lack detailed clinical background information and patient baseline characteristics, making it difficult to accurately assess causality. Additionally, the quality of reports varies, possibly containing duplicate, incomplete, or erroneous information, further affecting the reliability of the data. Moreover, the lack of accurate population data makes it challenging to calculate the incidence rate of adverse events. Nevertheless, FAERS remains a valuable pharmacovigilance tool, offering large-scale, real-time capability for detecting safety signals, promptly capturing rare or emerging adverse reactions, and providing crucial leads for regulatory decisions and further research.

## Conclusion

In this study, we comprehensively evaluated drugs associated with AIH by mining adverse reaction reports related to AIH in the FAERS database. We found that antibiotics, statins, and biologics are associated with AIH, which is consistent with previous published studies. Additionally, we identified certain drugs that have not been previously reported but possess potential risks of inducing AIH. Our study results provide important references for clinical formulation of strategies to manage adverse drug events.
